# Application of convolutional neural networks for histopathological diagnosis of feline low-grade T-cell lymphoma and lymphoplasmacytic enteritis in intestinal biopsies

**DOI:** 10.3389/fvets.2026.1851689

**Published:** 2026-06-16

**Authors:** Sabrina Vanessa Patrizia Defourny, Silvia Sabattini, Miranda Wright, Eugenio Faroni, Hayley Crosby-Durrani, Emanuele Ricci, Guido Rocchigiani, Antonella Rigillo, Laura Marconato, Marco Pietra, Antonio Petrini, Giuliano Bettini, Lorenzo Ressel

**Affiliations:** 1 Istituto Zooprofilattico Sperimentale dell’Abruzzo e del Molise, Teramo, Italy; 2 Department of Veterinary Medical Sciences, University of Bologna, Bologna, Italy; 3 Department of Veterinary Anatomy Physiology and Pathology, University of Liverpool, Neston, United Kingdom; 4 I-vet Diagnostics, Cellatica, Brescia, Italy

**Keywords:** artificial intelligence diagnostics, convolutional neural network, digital pathology, feline chronic enteropathy, low-grade T-cell lymphoma, lymphoplasmacytic enteritis

## Abstract

**Introduction:**

Low-grade intestinal T-cell lymphoma (LGITL) and lymphoplasmacytic enteritis (LPE) are common causes of feline chronic enteropathy. Although histopathology is the diagnostic gold standard, distinguishing between LGITL and LPE is challenging due to overlapping morphological features and high interobserver variability. Convolutional Neural Networks (CNNs) may improve diagnostic consistency and accuracy.

**Methods:**

This proof-of-concept study retrospectively analysed 161 formalin-fixed, paraffin-embedded endoscopic intestinal biopsies from cats diagnosed with LGITL or LPE. Samples were blindly re-evaluated according to standard guidelines using haematoxylin-eosin (H&E) and immunohistochemistry (IHC) by two board-certified veterinary pathologists; discordant cases were excluded. An InceptionV3 CNN, trained via transfer learning, was applied to 8,026 manually selected image tiles (1,024 × 1,024 pixels, RGB) obtained from selected digitized H&E-stained sections. Training included tile balancing and image augmentation, and the network used a 5-fold cross-validation strategy. The same cases were independently reviewed by three board-certified pathologists for comparison.

**Results:**

The final dataset comprised 142 cases, including 104 LPE and 38 LGITL. A test set of 23 cases was classified by the CNN, which generated a final diagnosis based on majority tile vote. The CNN achieved an average tile-level accuracy of 85.3% in cross-validation, focusing attention on lymphocyte-rich areas, as indicated by Grad-CAM analysis. At the case level, using a tile majority vote system, the CNN correctly diagnosed ~95% of 23 test cases in 1 min and 20 s, compared to pathologists’ average accuracy of ~85% in 9 min. Notably, the only case misclassified by the CNN was also misdiagnosed by two of the three pathologists.

**Discussion:**

These findings suggest that CNN-based analysis has strong potential as a diagnostic support tool for differentiating LGITL from LPE. Further model optimization and dataset expansion may enhance performance.

## 1 Introduction

Low-grade intestinal T-cell lymphoma (LGITL) and lymphoplasmacytic enteritis (LPE) are the most frequent diagnoses in cats with chronic gastrointestinal signs. Despite their distinct biological nature, these conditions often present with overlapping clinical signs and cannot be reliably distinguished through physical examination, blood tests, or diagnostic imaging (1). Due to shared morphological features and significant interobserver variability, even histopathological evaluation may sometimes yield inconclusive results (2). The distinction is particularly challenging when only endoscopic biopsies are available, as these samples are generally limited to mucosa and therefore preclude evaluation of deeper mural infiltration, which represents a more definitive histologic hallmark of lymphoma (1, 2). Immunohistochemistry (IHC) improves accuracy but overlap between LGITL and LPE may still result in diagnostic ambiguity.

From a clinical perspective, accurate and timely differentiation between LGITL and LPE is critical, as their management differs. LPE is usually treated primarily with corticosteroids, whereas LGITL requires the addition of chemotherapy with chlorambucil as first-line therapy. Additionally, the prognosis differs between the two conditions, with the latter associated with a poorer outcome. Consequently, misclassification may lead to inappropriate treatment and adversely affect patient outcomes.

Convolutional neural networks (CNNs), a class of deep learning models, can automatically extract relevant features from histological images and support diagnostic workflows (3). In human pathology, CNNs have achieved near-perfect accuracy in lymphoma classification across multiple studies (4–6), often matching or surpassing expert pathologists. In veterinary pathology, CNNs applications remain limited but promising. Haghofer et al. (7) introduced a modular deep learning workflow to classify canine lymphomas according to nuclear size on histology images, while Hubbard-Perez et al. (8) demonstrated that deep learning can differentiate hyperplastic lymph nodes from lymphomas and further efficiently subclassify different lymphoma histotypes. In feline gastrointestinal pathology, Wulcan et al. (9) developed an artificial intelligence (AI) model to quantify intraepithelial and lamina propria lymphocytes in intestinal biopsies, achieving high sensitivity. These findings indicate that CNNs can reliably extract histological features and improve reproducibility in otherwise subjective tasks. However, end-to-end classification of LGITL versus LPE in feline intestinal biopsies using AI remains unexplored.

The present study aims at evaluating the diagnostic performance of an InceptionV3 CNN trained on images from haematoxylin-eosin (H&E)–stained feline small intestine endoscopic biopsies to distinguish LGITL from LPE. Secondary objectives include comparing the model’s performance with that of pathologists, assessing prediction confidence, and evaluating the interpretability of model predictions using gradient-weighted class activation mapping (Grad-CAM).

## 2 Materials and methods

### 2.1 Case selection and histological evaluation

Archival formalin-fixed, paraffin-embedded feline small intestine endoscopic biopsy specimens diagnosed as either small cell lymphoma or enteritis were obtained from the Pathology Service of the Department of Veterinary Medical Sciences of the University of Bologna, Italy. Cases were identified by searching the institutional pathology database using keywords including “small cell lymphoma”, “small T-cell lymphoma”, “low-grade lymphoma”, “low grade T-cell lymphoma”, “mucosal lymphoma”, “lymphoplasmacytic”, “enteritis.” In all cases, standard upper and lower gastrointestinal endoscopic procedures were performed, with multiple biopsy specimens (typically 3–5 fragments) collected from each intestinal segment, including stomach, duodenum/jejunum, ileum, and colon. Cases were excluded if tissue quality was insufficient for meaningful histopathologic and immunohistochemical evaluation. Two board-certified veterinary pathologists (SS, AR; “ground truth pathologists”) independently reviewed all available H&E-stained sections along with serial sections immunolabelled for CD3 (T cell marker, Dako, Glostrup, Denmark) and CD20 (B-cell marker, Thermo Scientific, Waltham, MA, United States) to assign a new diagnosis according to current diagnostic gold standards (1). LGITL diagnoses were supported by histological features including monomorphic infiltration of small CD3-positive lymphocytes, intraepithelial lymphocyte crowding, architectural distortion, extension of infiltrates beyond the superficial lamina propria, and reduced inflammatory heterogeneity. Conversely, LPE cases were characterized by mixed inflammatory infiltrates including plasma cells, preservation of mucosal architecture, and lower degrees of epitheliotropism. All slide reviews were performed blinded to previous diagnoses, follow-up and anamnestic information. Only cases with concordant diagnoses of LGITL or LPE were included.

### 2.2 Histological image preparation

For each case, a diagnostic section was selected, corresponding to the H&E-stained section considered most representative by consensus between the two ground-truth pathologists, following review of all available sections and immunohistochemistry. The selected section was digitized as a whole-slide image (WSI) in RGB colour format using a high-throughput digital slide scanner (Aperio CS2 - Leica Biosystems) at 400x final magnification. WSIs were subdivided into non-overlapping 1,024 × 1,024-pixel tiles using ImageJ “Slide J” plugin (10) in tiff format. The tile size was chosen to be large enough to encompass relevant lesion context while remaining feasible for CNN input processing. Tiles were manually reviewed by two veterinary pathologists (SD, LR) to retain those containing ≥30% relevant tissue, while discarding those predominantly containing background, normal tissue, blur, or artifacts. All retained tiles were stored for input into the deep learning pipeline. To mitigate tile-count imbalance, we performed case-level tile balancing by randomly sampling from the manually curated tiles so that each case contributed a comparable number of tiles to the training set while maintaining an approximately equal tile representation across classes. After manual tile selection and case-level tile balancing, the final curated dataset comprised 8,026 image tiles, including 4,012 LPE tiles and 4,014 LGITL tiles.

To prevent data leakage between training and test set, case-level independence was maintained by splitting the dataset into five folds using a custom MATLAB routine (Supplementary Figure 1). All tiles from the same biopsy were assigned exclusively to a single fold, ensuring the CNN was trained on four folds (~80% of cases) and tested on the remaining fold (~20%). Prior to model training within each fold, image augmentation was applied to improve robustness using MATLAB (MathWorks, Natick, MA, United States) including X and Y axis flip and rotation.

### 2.3 CNN architecture and training

A CNN based on the InceptionV3 architecture was implemented using transfer learning in MATLAB R2023a (MathWorks, Natick, MA, United States) with the Deep Learning Toolbox and Neural Network Designer (11). InceptionV3 is a 48-layer convolutional neural network originally trained on ImageNet and designed to extract multi-scale image features through inception modules. The pretrained network was imported into Neural Network Designer, and the final classification layers were replaced with new task-specific layers for binary classification of the two diagnostic categories, LGITL and LPE. The final learnable layer was modified to match the number of output classes, followed by a softmax layer and a classification output layer. Each 1,024 × 1,024-pixel RGB histological tile was resized to 299 × 299 × 3 pixels to match the required input size of InceptionV3. Model training was formulated as a supervised binary classification task using the consensus diagnosis of the ground-truth pathologists as the class label. Five-fold cross-validation was used, with folds generated at case level to prevent data leakage; therefore, all tiles from the same biopsy were assigned exclusively to either the training or held-out test set within each fold. For each iteration, four folds were used for training and the remaining fold was used for testing. Transfer learning was performed by fine-tuning the modified InceptionV3 network on the histological tile dataset. Training used categorical cross-entropy loss and the Adam optimizer with *β*₁ = 0.9 and *β*₂ = 0.999 for 10 epochs. Image augmentation was applied only to training tiles and included random horizontal and vertical flipping and rotation, whereas no augmentation was applied to held-out test tiles or during inference. Training progress was monitored using accuracy and loss curves to assess learning behaviour and potential overfitting. The final trained network from each fold was saved and subsequently applied to the corresponding held-out test fold to obtain tile-level class predictions and class probabilities. Training was performed on a workstation equipped with two NVIDIA Quadro RTX 6000 GPUs, with a combined VRAM of 48 GB. Tile-level predictions were subsequently aggregated at case level using a majority-vote approach, whereby the final case diagnosis was assigned according to the class predicted in the majority of tiles from that biopsy.

### 2.4 Evaluation and performance comparison

Each trained fold model was first evaluated on its held-out test tiles to measure tile-level classification performance. For every tile, the CNN predicted a class label (LGITL or LPE) with an associated probability, which was compared to the ground truth pathologists’ diagnosis to compute standard performance metrics. Confusion matrices were obtained and examined to identify any systematic misclassification patterns. For tile-level cross-validation results, performance variability across the five folds was summarized as mean ± standard deviation (SD). Metrics calculated for each fold included accuracy, precision, recall, and F1-score, for both training and held-out test data.

Grad-CAM was applied using a custom code (Supplementary Figure 2) to a randomly selected subset of test tiles from the two classes, obtained with the RANDBETWEEN function in Excel software, to visualize regions most influential for the CNN’s predictions. Heatmaps were overlaid on the original images and reviewed by two pathologists (LR, SD) to confirm that the network focused on histologically relevant areas (e.g., lymphocyte-rich areas) rather than artifacts (12).

Tile-level predictions were then aggregated using a majority-vote scheme to assign an overall diagnosis per biopsy, as commonly applied in WSI-based deep learning studies (13).

To evaluate diagnostic discrimination beyond the predefined majority-vote threshold, case-level receiver operating characteristic (ROC) analyses were performed. Because the final diagnostic output of the CNN was assigned at biopsy level, ROC/AUC analyses were conducted at case level rather than tile level. For each case, the proportion of tiles classified by the CNN as LGITL and the proportion classified as LPE were used as continuous diagnostic scores.

**Figure 1:**
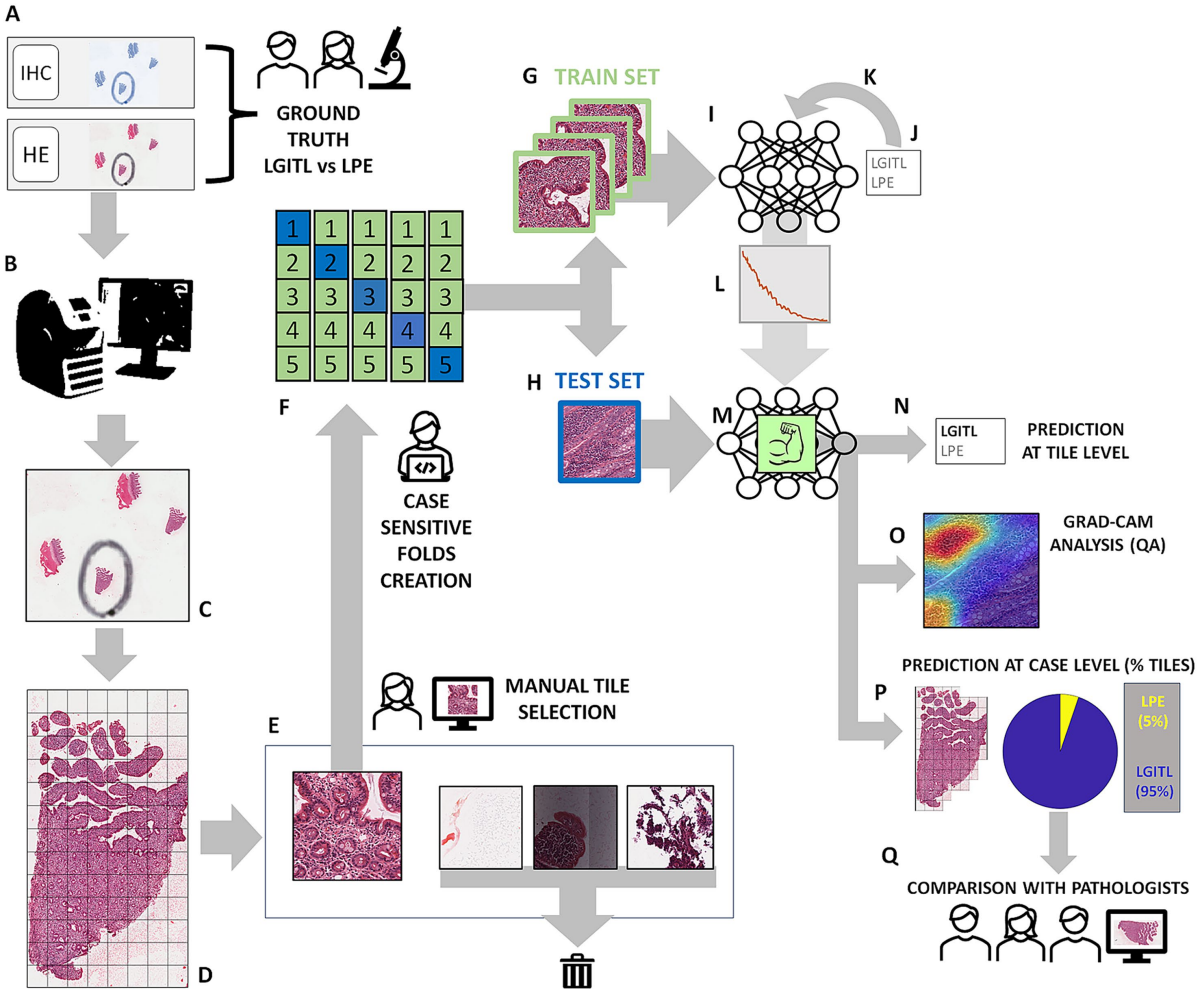
Workflow for training and testing the convolutional neural network (CNN) to classify LGITL and LPE. The process starts with H&E and IHC slides from cases diagnosed independently by two pathologists using established criteria; discordant cases are excluded **(A)**. H&E slides, with the diagnostic region used for consensus marked, are scanned with a digital slide scanner **(B)** to generate a whole-slide image (WSI) **(C)**. The WSI is divided into 1,024 × 1,024 pixel RGB tiles **(D)**. Tiles are manually curated: those representative of the lesions are retained, whereas background tiles or tiles containing artefacts are discarded **(E)**. Custom code divides tiles into five folds, ensuring that tiles from the same case are kept within a single fold **(F)**. Four folds are used for training (G) and the remaining fold for testing **(H)**. During training, tiles from the training set are fed into the CNN **(I)**, which outputs class probabilities **(J)**; prediction error is then used to update network weights via backpropagation **(K)** for the predefined training schedule **(L)**, yielding a trained network **(M)**. The trained network is then used to: (1) classify previously unseen test-set tiles at tile level **(N)**; (2) generate Grad-CAM heatmaps to highlight image regions contributing most to the classification decision (quality control/interpretability) **(O)**; and (3) produce a case-level classification by majority vote across tiles from the same case **(P)**. Case-level predictions are compared with pathologists’ diagnoses on the diagnostic WSI **(Q)**.

To contextualize the CNN’s performance, three board-certified veterinary pathologists (HCD, ER, GR; “test pathologists”) independently reviewed all the WSI H&E test slides, blinded to prior diagnoses and IHC results, focusing on the section deemed diagnostic by the ground-truth pathologists and rendered a diagnosis of LGITL or LPE while the time needed to reach a decision was monitored for each case by a human operator (LR). For timing, each pathologist was assessed independently using the same PC, monitor, and digital slide viewer; for each case the timer was started when the WSI was presented to the pathologist, stopped when they verbally confirmed a diagnosis, and then restarted for the next case after the operator loaded the subsequent WSI, repeating this sequence until all cases were completed. Their results were compared to the ground truth diagnosis as the gold standard, and accuracy, sensitivity, and specificity were calculated. The CNN’s case-level accuracy was compared with that of the test pathologists. Instances of disagreement were examined by recording cases in which the CNN correctly classified LGITL or LPE whereas one or more pathologists did not, and vice versa. Agreement was assessed with Cohen’s kappa, and paired differences between CNN and pathologists were evaluated using McNemar’s test. To explore diagnostic difficulty, the percentage of correctly classified tiles per case (AI confidence) was correlated with the time pathologists required to reach a diagnosis. Statistical analyses were performed in Python using scikit-learn (Cohen’s kappa), statsmodels (McNemar’s test), and SciPy (Pearson’s correlation), with *p* < 0.05 considered significant. Case-level ROC analyses were performed in MATLAB using the “perfcurve” function. A scheme exemplifying the experimental design is presented in Figure 1.

## 3 Results

### 3.1 Case selection and cohort characteristics

A total of 161 endoscopic small intestinal biopsies were initially retrieved. After blinded revision, 19 discordant cases (11.8%) were excluded, leaving 142 concordant diagnoses: 104 (73.2%) LPE and 38 (26.8%) LGITL.

### 3.2 CNN training and test performance

Across all networks, training accuracy ranged from 97.00% (Network 1) to 98.19% (Network 3). The highest F1-score during training was observed in Network 3 (98.27%), while Network 4 achieved the highest recall (98.88%). Across the five folds, training performance was consistently high, with mean accuracy of 97.62 ± 0.57%, precision of 97.85 ± 0.72%, recall of 97.39 ± 1.19%, and F1-score of 97.62 ± 0.57%.

Testing performance showed more variability, with mean accuracy of 85.30 ± 1.28%, precision of 83.40 ± 6.55%, recall of 88.50 ± 2.36%, and F1-score of 85.69 ± 2.39%. Accuracy rangied from 83.20% (Network 2) to 86.32% (Networks 3 and 5). Network 5 achieved the best general performance, with a test F1-score of 88.89% and the highest precision (91.21%). Conversely, Network 2, while strong in training, exhibited a drop in test performance, likely due to mild overfitting. Average test results were as follows: accuracy 85.30%, precision 83.40%, recall 88.50%, and F1-score 85.69%. Network 3 was selected for subsequent analyses as it represented an average performing network among the 5 tested. A summary of the metrics is presented in Table 1. Confusion matrices for the different networks are presented in Supplementary Figure 3.

**Table 1:** Summary of train and test metrics for tile-based image recognition.

Network	Train accuracy	Train precision	Train recall	Train F1-score	Test accuracy	Test precision	Test recall	Test F1-score
Network 1	0.97	0.97	0.97	0.97	0.85	0.81	0.90	0.85
Network 2	0.97	0.99	0.96	0.97	0.83	0.77	0.90	0.83
Network 3	0.98	0.99	0.98	0.98	0.86	0.78	0.90	0.84
Network 4	0.98	0.97	0.99	0.98	0.85	0.90	0.85	0.87
Network 5	0.98	0.97	0.98	0.98	0.86	0.91	0.87	0.89
Mean ± SD	0.976 ± 0.006	0.979 ± 0.007	0.974 ± 0.012	0.976 ± 0.006	0.853 ± 0.013	0.834 ± 0.066	0.885 ± 0.024	0.857 ± 0.024

### 3.3 Grad-CAM visualization

**Figure 2:**
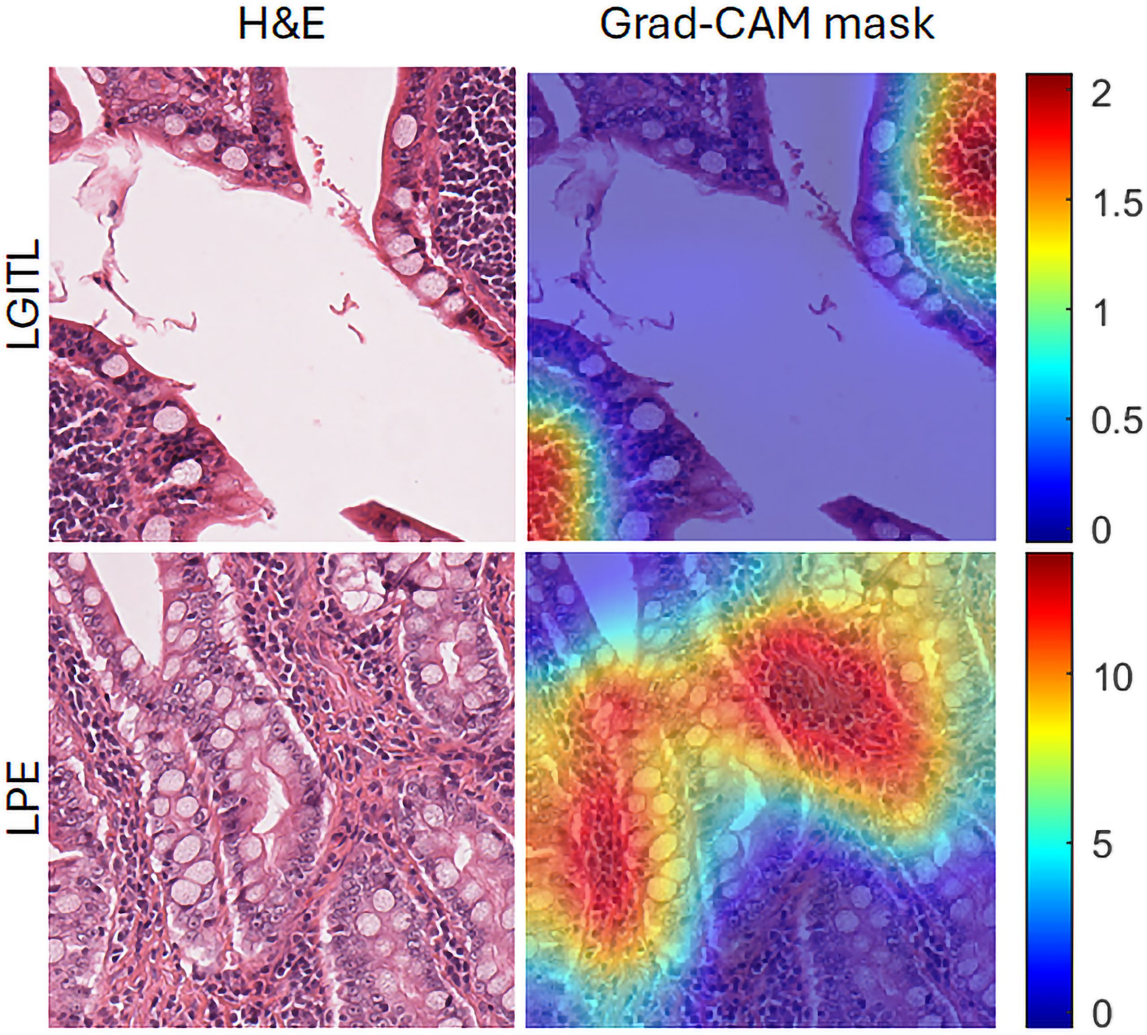
Examples of LGITL and LPE tiles and relative Grad-CAM heatmaps indicating class-discriminative regions learned by the CNN. Colors represent relative importance scores (unitless), derived from the gradient-weighted activations in the final convolutional layer. Warmer colors indicate higher contribution to the predicted class. Heatmap values are shown in raw form and vary in scale depending on image content and network gradients.

Grad-CAM heatmaps were generated for the tiles obtained from a test set of 23 cases. The regions of highest attention consistently overlapped with lymphocyte-rich areas in both LGITL and LPE. No high-attention regions were observed on background or artefactual areas. Representative examples are shown in Figure 2.

### 3.4 Case-level diagnosis by majority voting and comparison with test pathologists

**Figure 3:**
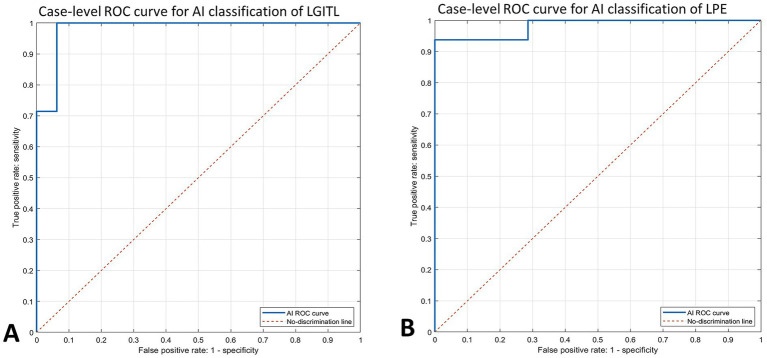
Case-level receiver operating characteristic (ROC) curves for CNN classification of LIGTL and LPE. ROC analysis was performed at biopsy level using the proportion of tiles assigned to each diagnostic category as the continuous diagnostic score. **(A)** ROC curve using LIGTL as the target class and the case-level LIGTL tile score as the diagnostic score. **(B)** ROC curve using LPE as the target class and the case-level LPE tile score as the diagnostic score. In both analyses, the CNN achieved an AUC of 0.982, indicating excellent discrimination between LIGTL and LPE. The dashed diagonal line represents the no-discrimination reference line.

In the majority-vote aggregation strategy employed on Network 3, a total of 23 cases not used for training were evaluated. For each case, a variable number of annotated image tiles were analysed, ranging from 8 to 357 tiles per case, with an average of 66.74 tiles. Out of the 23 cases, 22 (95.65%) were correctly classified at the case level. The percentage of correctly classified tiles per case ranged from 62.50 to 100.00%, with an average of 89.32%. The single misclassified case showed 19.61% correctly predicted tiles. At the predefined majority-vote threshold of 0.50, the case-level confusion matrix included 7 true LGITL cases correctly classified as LGITL, 15 true LPE cases correctly classified as LPE, 1 LPE case incorrectly classified as LGITL, and no LGITL cases incorrectly classified as LPE. This corresponded to an accuracy of 95.65% (22/23; 95% CI: 78.05–99.89), sensitivity for LGITL of 100% (7/7; 95% CI: 59.04–100), specificity of 93.75% (15/16; 95% CI: 69.77–99.84), precision for LGITL of 87.50% (7/8; 95% CI: 47.35–99.68), negative predictive value of 100% (15/15; 95% CI: 78.20–100), and F1-score of 0.933. Case-level ROC analyses were performed using both diagnostic categories as target classes. When LGITL or LPE were used as the target class and the proportion of tiles classified as LGITL or LPE, respectively, the CNN achieved an AUC of 0.982 (Figure 3). At the predefined threshold of 0.50, the performance metrics were those reported above.

For the complementary ROC analysis involving LPE as the target class, at the 0.50 threshold, sensitivity for LPE was 93.75% (15/16; 95% CI: 69.77–99.84), specificity was 100% (7/7; 95% CI: 59.04–100), precision for LPE was 100% (15/15; 95% CI: 78.20–100), negative predictive value was 87.50% (7/8; 95% CI: 47.35–99.68), and F1-score was 0.968.

On the same 23 cases, diagnostic accuracies of the three test pathologists were 86.96, 82.61, and 86.96%, respectively (mean 85.5%). The CNN required an average of 3.50 s per case, compared with 17.49, 26.65, and 25.96 s for the pathologists. Overall, the 23 cases were diagnosed in 80.40 and 537.40 s by the AI and the pathologists (on average), respectively. Based on the total number of tiles analysed, the processing speed of the CNN was 0.05 s per tile, with case-level inference times ranging from 0.42 to 18.72 s.

Cohen’s kappa values for agreement between CNN and test pathologists were *κ* = 0.82 (strong agreement) for pathologist 1, κ = 0.56 (moderate agreement) for pathologist 2, and κ = 0.82 (strong agreement) for pathologist 3. Notably, the single case misclassified by the CNN was also misclassified by two of the three test pathologists. McNemar’s exact test showed no significant differences between CNN and test pathologists (AI vs. pathologist 1, *p* = 0.50; AI vs. pathologist 2, *p* = 0.38; AI vs. pathologist 3, *p* = 0.50). Pearson’s correlation analysis showed weak, non-significant, negative associations between CNN classification accuracy and the time taken by test pathologists to reach a diagnosis. The results were as follows: pathologist 1: *r* = −0.13 (*p* = 0.57); pathologist 2: *r* = −0.15 (*p* = 0.50); pathologist 3: *r* = −0.27 (*p* = 0.22); Average diagnostic time: *r* = −0.23 (*p* = 0.29).

## 4 Discussion

In this study, we developed and validated a deep CNN proof-of-concept using InceptionV3 transfer learning to distinguish between LGITL and LPE in cats. Using 5-fold cross-validation on manually-curated biopsy tiles and majority voting for slide-level diagnosis, the model’s performance was compared with that of board-certified veterinary pathologists. Grad-CAM was employed for interpretability of the AI decision process. The CNN achieved case-level accuracy comparable to human experts while significantly reducing diagnostic time.

A key strength of this study is the use of a high-confidence, independent ground truth. Unlike many AI studies that rely on pathologist annotations on H&E slides, our cases were originally diagnosed by consensus using both H&E morphology and IHC according to current guidelines (1). This approach minimizes circular validation and avoids bias introduced when algorithms or observers are assessed against the same subjective HE-based interpretation that guided their predictions. Instead, it provides a higher-confidence gold standard/reference standard that is independent of the H&E-only test reads and which more accurately reflects true biological status. Consequently, agreement or disagreement with this composite reference reflects genuine diagnostic capability rather than concordance with human opinion.

Another study applying AI to quantify lymphocytes across entire WSIs in feline intestinal biopsies achieved high sensitivity but only moderate positive predictive value (~57%) due to false positives and overlapping lymphocyte counts between LPE and lymphoma (9). In contrast, our CNN appears to capture additional contextual information, such as lymphocyte clustering and tissue architecture, enabling a holistic pattern recognition that accurately distinguishes LGITL from LPE. Combining quantitative lymphocyte detection with CNN-based tissue pattern analysis could represent a promising integrated workflow for future studies. Our results demonstrate that a deep CNN can effectively distinguish feline LGITL from LPE on H&E slides, achieving performance comparable to experienced pathologists while dramatically reducing interpretation time. Training metrics were very high (>97% accuracy), and although test tile-level accuracy averaged ~85% across folds, the model generalized well overall. This moderate drop from training to testing is expected given the limited dataset and subtle morphological overlap between LGITL and LPE. Importantly, by aggregating tile predictions at the case level, the CNN’s slide-level accuracy rose to 96% (22/23 cases correct), indicating that the voting scheme captured the overall diagnosis more reliably than any single image patch. Using this approach, rather than relying on a single small field as in previous studies (8) the diagnosis emerges from a consensus of multiple fields of view (or tiles for AI), akin to how a pathologist scans different regions of a biopsy before emitting a judgement. Majority voting proved to be a simple yet effective ensemble method for this problem. However, incorporating an uncertainty measure or confidence threshold would be prudent. Our current model produces a binary case-level prediction, although each case has an associated probability reflecting the results across its tiles. Using majority voting, even a 51% probability of LGITL results in a lymphoma classification. In a clinical setting, when the vote is close, the AI should express low confidence and suggest deferring to human judgment. Additionally, in practice, pathologists often base their diagnosis on particularly meaningful areas of a slide, even if they do not represent the majority of the tissue. Although our approach minimized that bias focusing both the CNN and the test pathologist on a single section which was considered the diagnostic one, nevertheless this approach can limit the reasoning behind majority voting, since all tiles are treated equally in the final decision.

At the case level, as previously mentioned, the CNN’s model achieved 96% case accuracy (1 wrong out of 23), while individual pathologists ranged from 83 to 87% on H&E alone, with no significant difference. These results align with the growing body of literature suggesting AI can match specialist-level accuracy in pathology classification (14, 15). Recent large-scale reviews in computational pathology have highlighted that deep learning models can achieve diagnostic performances comparable to expert pathologists across multiple histopathological tasks, particularly when integrated into human-AI collaborative workflows (16, 17). High concordance was observed between the CNN’s diagnoses and those of the pathologists (Cohen’s *κ* = 0.82 with two of three observers, and moderate agreement with the third), indicating that AI generally agreed with pathologist judgment, which supports its potential use as a diagnostic co-pilot or automatic triage system. Such an approach could also serve as an artificial second opinion: a disagreement between pathologist and AI could prompt to additional scrutiny or ancillary testing. The single misdiagnosed case by the model had fewer than 20% of tiles correctly classified, and the same case was misdiagnosed by two out of three pathologists, suggesting that the majority of histologic features on this slide genuinely favored an incorrect impression, both in humans and AI. This highlights that borderline cases on H&E may be inherently ambiguous, necessitating additional tests.

A clear benefit of the CNN approach is speed. Our model processed an entire case (tens to hundreds of tiles) in an average of ~3.5 s – roughly 5–10 × faster than the pathologists. In practical terms, the AI can render a preliminary interpretation almost immediately once the slide is scanned. This finding is in line with the promise that AI can automate tedious aspects of slide reading and accelerate the diagnostic workflow (18) potentially delegating the initial screening to AI while the pathologist reviews the algorithm’s findings. Recent reviews in digital pathology further emphasized that AI-based tools are particularly valuable for reducing repetitive screening workload, improving workflow efficiency, and prioritizing diagnostically challenging cases for expert review (17, 19). Following this principle, recent work showed that pathologists were over 60% faster when assisted by AI, without loss of accuracy (20), further suggesting that the most realistic role of AI in this context is as a co-pilot rather than an autonomous diagnostician, intended to assist and augment pathologists, instead of replacing them (20). A workflow such as the one presented here could reduce time spent on straightforward cases, allowing human effort to focus on cases with mixed features or where AI and human assessment diverge.

To understand which areas the CNN relied on for diagnosis, Grad-CAM heatmaps were evaluated. Grad-CAM heatmaps indicated that the CNN’s predictions were driven by histologically relevant mucosal regions rather than background or artefact. However, this finding should not be interpreted as evidence that the model relied simply on lymphocyte density, since both LGITL and LPE are lymphocyte-dominant conditions. In addition, Grad-CAM does not permit precise identification of individual cellular features used by the network. Given that the tile size was selected to preserve tissue context, the model may have learned broader patterns such as the spatial organization of lymphocytes, their relationship with epithelium and crypts, mucosal architectural changes, clustering, and inflammatory heterogeneity. These interpretations remain speculative but are consistent with the contextual information pathologists use when distinguishing LGITL from LPE. Anyway Grad-CAM analysis provided confidence that it learned legitimate histopathological features rather than spurious correlates. As explainability becomes increasingly recognized as an essential criterion for clinical AI applications, Grad-CAM visualization offers a window into the CNN’s “black-box” decision-making, helping to build trust and transparency (21). In challenging differential diagnoses such as LGITL versus LPE, Grad-CAM could also guide pathologists to areas of interest, suggesting that future synergy between AI and human insight may become a cornerstone of effective AI-assisted diagnostics.

Despite these promising results, several limitations must be acknowledged. First, the dataset was derived from a single institution, with slides prepared under consistent protocols and scanned on the same platform. AI models are sensitive to domain shifts, such as variations in staining intensity, section thickness, and scanning equipment, which can degrade performance when applied to external data. Similar issues have been reported in multi-center lymphoma studies, where models achieving near-perfect internal accuracy initially dropped on external cohorts due to technical differences (5). To ensure generalizability, models should be tested on independent cohorts, and additional local training may be necessary. Site-specific adaptation strategies have been successfully applied in other medical imaging fields, allowing robust performance without requiring global standardization (22, 23). In our study, even though each fold in the 5-fold validation was held out from training, the absence of a fully independent external test set remains a limitation. Cross-validation provides an internal estimate of generalisation by repeatedly training on disjoint subsets and evaluating on unseen cases; in this sense, the results for the held-out fold are methodologically valid as an assessment on genuinely unseen material. However, because model development was performed within the same overall dataset, performance estimates may still be optimistic and may not fully capture variability due to differences in case mix, staining/scan conditions, and institutional workflows.

Second, our sample size was relatively small for a deep learning task. Although five-fold cross-validation and transfer learning were used to maximize the data utility, a larger dataset might capture a wider range of histologic variation. LGITL in cats can present with subtle differences from case to case such as degree of epitheliotropism and cell morphology while LPE cases vary in severity and density of the infiltrate. A bigger sample could certainly help the CNN learn additional features.

Third, a further relevant limitation is that we excluded cases with inter-observer discordance to ensure a more robust ground truth as our label accuracy relied entirely on the fidelity of established morphological (and immunophenotypic) criteria without additional data; while this concordance-based approach reduces mislabeling risk for model training and evaluation, it also removes the most borderline clinically relevant cases and may therefore overestimate performance in real-world diagnostic practice. Future studies should specifically incorporate diagnostically ambiguous and discordant cases to better reflect the full spectrum of pathology practice. In such scenarios, probabilistic or uncertainty-aware AI approaches may be particularly valuable, allowing the model to express low-confidence predictions and potentially flag borderline cases requiring additional human review or ancillary testing rather than forcing a binary classification.

Fourth, the classification here was binary (LGITL vs. LPE), which means the model was not exposed to any category outside of the two considered. In the presence of unrelated disease, the model would still force a prediction of LPE or LGITL, which could be misleading. Future work should broaden the model to additional classes or incorporate outlier detection to flag cases requiring careful review.

In summary, this study highlights the potential of deep learning as a supportive tool in veterinary diagnostic histopathology. By using a majority-vote system to aggregate tile predictions, our CNN resulted in accurate case-level diagnoses rather than fragmented tile opinions. The integration of Grad-CAM explainability ensured that the model’s decision process remains transparent and grounded in genuine histologic features, a crucial factor for building clinician confidence in AI-assisted diagnosis. We showed that this approach does not significantly outperform specialists in accuracy and its primary advantage lies in efficiency (time saving) suggesting it could act as a rapid screener, digital assistant or training tool to pathologists. In this study however, the reported CNN timing reflected only the image inference and case-level aggregation step after digitized images and selected tiles were already available. It therefore does not represent the full duration of a complete digital pathology workflow. With further refinement, expansion to multi-institutional data, and integration into workflow, AI systems such as this one could significantly enhance diagnostic throughput and consistency. Our findings contribute to the growing field of digital veterinary pathology demonstrating that in a challenging diagnostic scenario, an AI model can reliably mimic expert-level diagnoses with relevant time-saving improvement. As a proof-of-concept in a single-centre, manually curated tile workflow, external validation and expansion to uncurated WSIs are required before clinical deployment.

## Data Availability

The datasets presented in this study can be found in online repositories. The names of the repository/repositories and accession number(s) can be found at: Zenodo repository [www.zenodo.org] with DOI: 10.5281/zenodo.19295779. The dataset is viewable at: www.zenodo.org/records/19295779.
