# Supplementary material for: Application of convolutional neural networks for histopathological diagnosis of feline low-grade T-cell lymphoma and lymphoplasmacytic enteritis in intestinal biopsies
Source: Front Vet Sci. 2026 Jun 16;13:1851689. doi: 10.3389/fvets.2026.1851689 (PMC13314416; doi:10.3389/fvets.2026.1851689)

Supplementary Material

Supplementary Figure 1. Code used for generating the folds.


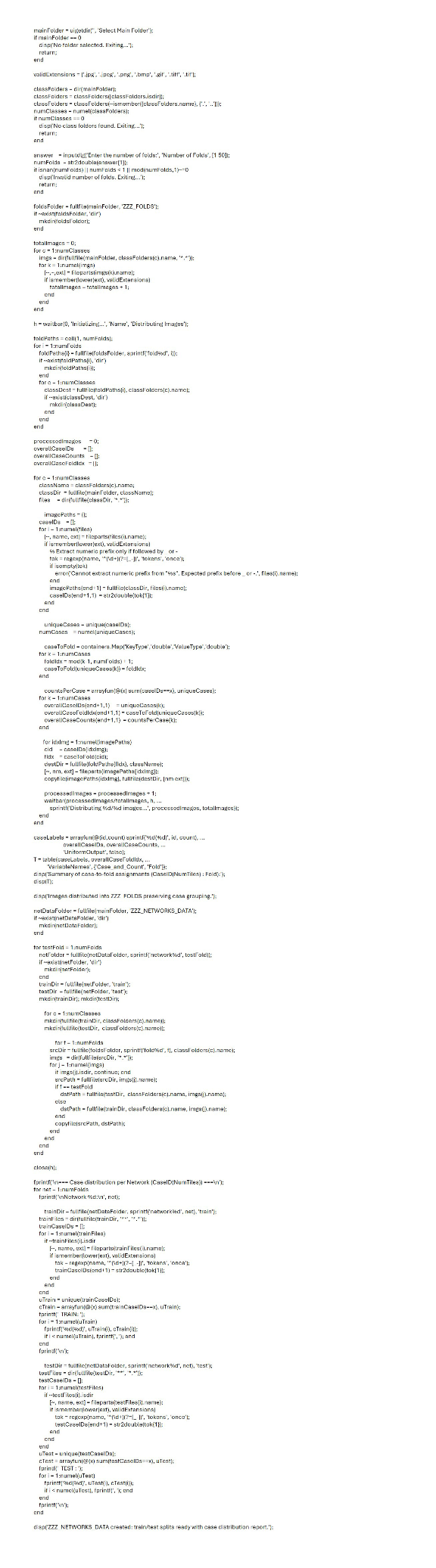


**Supplementary Figure 2.** Code used for Grad-CAM


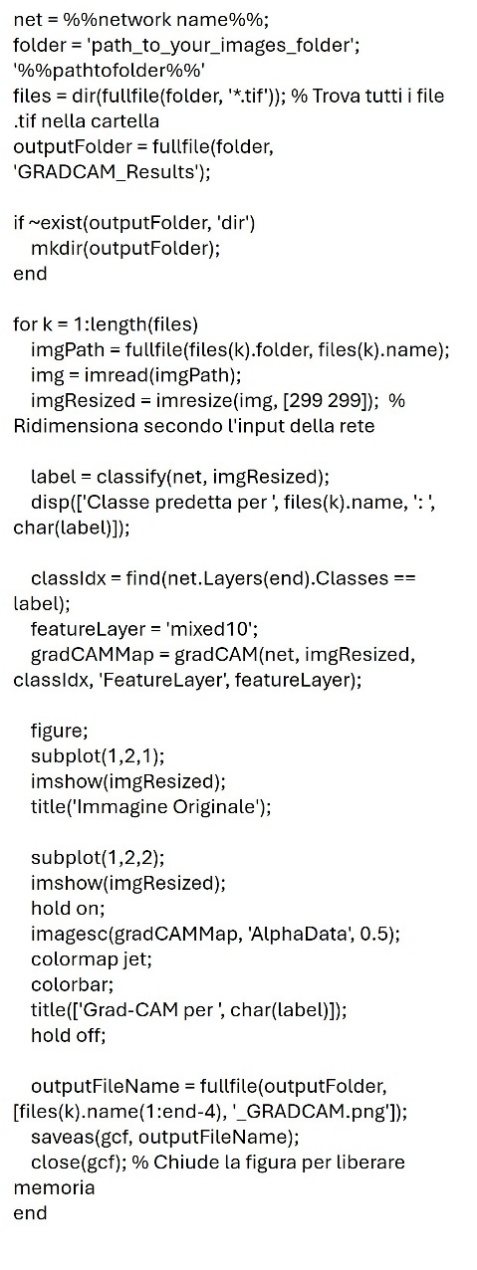


**Supplementary Figure 3.** Confusion matrices of Training and Test sets for the 5 different folds.


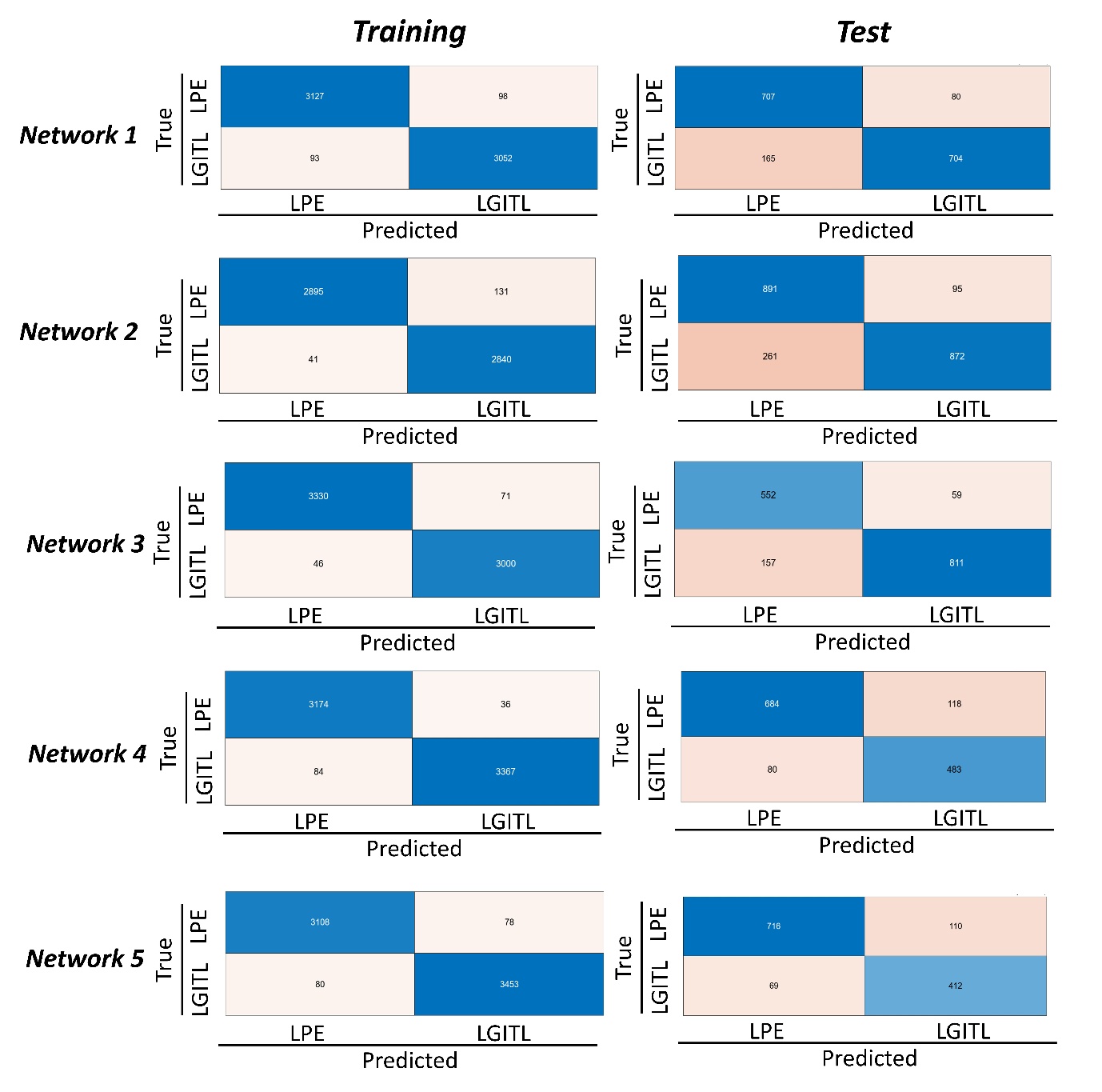

Supplement: Supplementary file 1 [file Table_1.docx]
